# Analysis of Modified Nucleotide Aptamer Library Generated by Thermophilic DNA Polymerases

**DOI:** 10.1002/cbic.202000236

**Published:** 2020-07-14

**Authors:** Krisztina Percze, Tamás Mészáros

**Affiliations:** ^1^ Department of Medical Chemistry Molecular Biology and Pathobiochemistry Semmelweis University 1094 Budapest Tűzoltó u. 37–47 Ungarn

**Keywords:** aptamers, base-modified nucleotides, DNA polymerase, NGS analysis, polymerase chain reaction

## Abstract

One of the pivotal steps in aptamer selection is the amplification of target‐specific oligonucleotides by thermophilic DNA polymerases; it can be a challenging task if nucleic acids possessing modified nucleotides are to be amplified. Hence, the identification of compatible DNA polymerase and modified nucleotide pairs is necessary for effective selection of aptamers with unnatural nucleotides. We present an in‐depth study of using 5‐indolyl‐AA‐dUTP (TAdUTP) to generate oligonucleotide libraries for aptamer selection. We found that, among the eight studied DNA polymerases, only Vent(exo‐) and KOD XL are capable of adapting TAdUTP, and that replacing dTTP did not have a significant effect on the productivity of KOD XL. We demonstrated that water‐in‐oil emulsion PCR is suitable for the generation of aptamer libraries of modified nucleotides. Finally, high‐throughput sequence analysis showed that neither the error rate nor the PCR bias was significantly affected by using TAdUTP. In summary, we propose that KOD XL and TAdUTP could be effectively used for aptamer selection without distorting the sequence space of random oligonucleotide libraries.

## Graphical abstract

**To select aptamers with modified nucleotides**, the appropriate DNA polymerase must be designated. We studied eight polymerases and demonstrated that incorporating a tryptophan‐like nucleotide into aptamer libraries by emulsion PCR and KOD XL had an insignificant effect on the PCR bias and did not distort the sequence space, thus making this approach the method of choice.

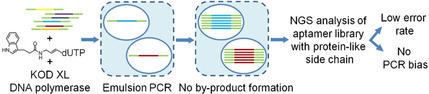

## Introduction

Aptamers are short, single‐stranded oligonucleotides of elaborate spatial structures that can bind to their target with outstanding specificity and high affinity. Thousands of publications have attested that aptamers are promising candidates for receptors of next‐generation diagnostic devices and therapeutic applications, but only a very limited number of aptamers have made their way into routine use.^1^ The unexpectedly low practical impact of aptamers could be partially explained by the inherent shortcomings of oligonucleotides composed of natural bases.^2^

The first aptamers were selected for a bacteriophage polymerase and organic dyes from random RNA oligonucleotide libraries by an iterative process known as SELEX.^3^, ^4^ Due to the assumption that the conformational flexibility of RNA oligonucleotides is higher than those of DNAs, RNA libraries remained to be preferred at the dawn of aptamer selection.^5^ Currently, most laboratories adapt DNA as the oligonucleotide library of selection because it has become evident that the success rate of SELEX is similar whether RNA or DNA is used.^6^ Although moving from RNA to DNA made the SELEX more straightforward and somewhat alleviated the instability issue of aptamers, only approximately 30 % of the selection process results in functional aptamers of practical significance, and the degradation of DNA aptamers by omnipresent DNases still limits their routine use.^5^

A battery of unnatural, chemically modified nucleotides have been used to improve the efficiency of SELEX and increase the endurance of aptamers in the prevailing environmental and physiological conditions. The first modified nucleotide containing aptamers were generated by the post‐SELEX approach that is aptamers were first isolated by using natural RNA or DNA libraries and then the unnatural nucleotides were incorporated at given positions during the chemical synthesis of oligonucleotides.^7^ History of the first commercialised aptamer‐based drug illustrates the shortcomings of this method. The lead molecule of pegaptanib was isolated by using a 2’‐F ribose‐modified RNA library, and the selection was followed by the insertion of further modified nucleotides in various locations and individual characterisation of each aptamer candidates that dramatically increased the cost and time demand of functional aptamer generation.^8^ In order to evade the post‐SELEX, oligonucleotide libraries of nonstandard nucleotides have been created either by directly introducing the modified nucleotide during the synthesis or by post‐synthetic addition of “clickable” nucleotides.^9^ These modifications could be divided into three broad categories according to the involved component of nucleotides, that is, sugar, phosphate, and base modifications.^2^ Dozens of unnatural oligonucleotides have been used with diverse success and according to the published data, introduction of hydrophobic and aromatic functional groups at positions oriented away from the hydrogen bonding sides of the bases, that is, the 5‐position of pyrimidines and the 8‐position of purines has the most significant effect on the success rate of aptamer selection.^7^

Based on this observation, a new class of aptamers with protein‐like side chains at the 5‐position of deoxyuridine has been developed. These so‐called slow off‐rate modified aptamers (SOMAmers) were successfully selected even for proteins that failed SELEX using an unmodified DNA library and most of them demonstrated sub‐nanomolar *K*_d_ values.^5^ Although the initial modified nucleotide holding library of aptamer selection could be produced by solid‐phase synthesis and by click chemistry, the SELEX essentially relies on enzymatic amplification of target selective oligonucleotides. Hence, the specific polymerases that are capable of incorporating the given non‐standard dNTP have to be identified prior to SELEX. Different commercial DNA polymerases have been studied to evaluate their capacity for using various modified nucleotides as substrates of DNA polymerisation.^10^ These studies demonstrated that family B polymerases are superior to the members of family A, both in terms of effectivity and accepting a more extended repertoire of unnatural nucleotides.^11^, ^12^, ^13^ Although some of these polymerases were successfully applied to generate base‐modified aptamers, no data have been presented showing the effect of using modified nucleotides on the distortion of oligonucleotide libraries amplified by PCR yet.

**Figure 1:**
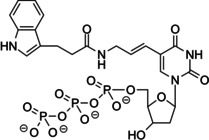
Introduction of 5‐indolyl‐AA‐dUTP (TAdUTP) confers tryptophan‐like chemical properties on DNA oligonucleotides.

Here, we present an in‐depth study of using 5‐indolyl‐AA‐dUTP (TAdUTP; Figure 1), an amino acid‐like side chain holding nucleotide for generating an oligonucleotide library for aptamer selection. We studied eight thermostable DNA polymerases and found that only Vent(exo‐) and KOD XL are capable of adapting the studied nucleotide. Next, the efficiency of TAdUTP incorporation was compared to those of dTTP by using individual oligonucleotide templates and we showed that replacement of dTTP did not have a significant effect on the productivity of KOD XL.

We also compared the products of open and water‐in‐oil emulsion PCR using an aptamer library as a template and demonstrated that the latter is suitable for the generation of aptamer libraries of the studied modified nucleotide. Finally, we employed high‐throughput sequencing to assess the error rate and PCR bias and found that none of them were significantly affected by the use of the unnatural nucleotide. Summarily, we present that KOD XL and TAdUTP could be effectively used for aptamer selection without distorting the sequence space of random oligonucleotide libraries.

## Results and Discussion

### Vent(exo‐) and KOD XL readily incorporate TAdUTP into oligonucleotides

**Figure 2:**
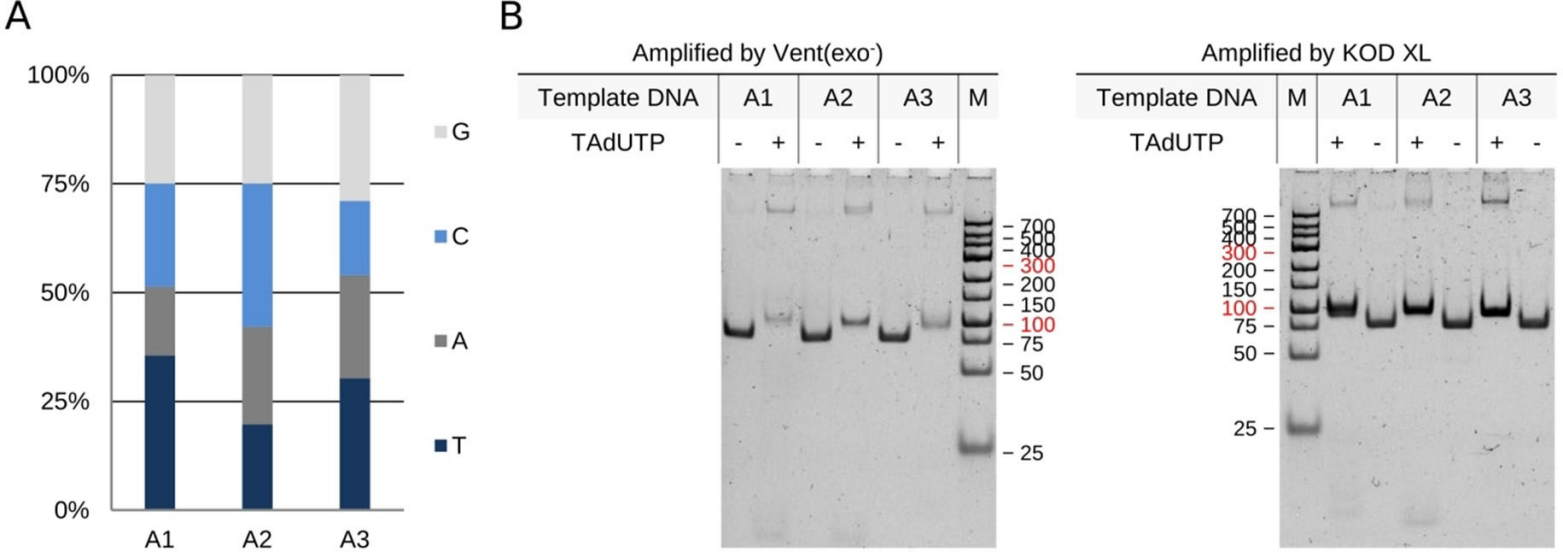
Amplification of aptamers with Vent(exo‐) and KOD XL by using dTTP or TAdUTP. A) Nucleotide ratios of template oligonucleotides. Aptamer no. 1 has the highest amount of thymine; aptamer no. 3 has the highest amount of thymine and adenine. B) 5 μL of KOD XL‐ and Vent(exo‐)‐catalysed reaction mixtures were separated by PAGE and visualised with GelGreen dye.

As previous publications demonstrated that using amino acid side chain holding oligonucleotide libraries increase the success rate of aptamer selection most significantly, we set out to systematically analyse the applicability of TAdUTP for generating aptamers of modified nucleotides. First, we aimed to identify thermostable polymerases that could replace thymines with TAdUTP during the polymerase chain reaction. We chose eight enzymes that were previously reported to accept modified nucleotides as substrates of DNA polymerisation and applied three oligonucleotides of different base composition as templates for PCR (Figure 2A, Table 1 and Table S1 in the Supporting Information). The accomplished amplification reactions demonstrated that none of the polymerases with 3’–5’ exonuclease activity produced any product when TAdUTP was provided instead of dTTP (Table 1 and Figure S1). Interestingly, the 3’‐5’ exonuclease activity deficient 9°N variant, Therminator was also unable for incorporation of the studied unnatural nucleotide (Table 1 and Figure S1). In contrast to Therminator, both of the other two 3’–5’ exonuclease domain lacking enzymes, Vent(exo‐) and KOD XL synthesised DNA fragments of expected size even when dTTP was substituted with TAdUTP (Figure 2B). Although both of these enzymes accepted the modified nucleotide, KOD XL seems to be superior to Vent(exo‐) since the addition of the modified nucleotide did not affect its efficiency while an obviously lower yield was obtained when the reaction was catalysed by Vent(exo‐) (Figure 2B).

**Table 1:** Main characteristics of the studied thermophilic DNA polymerases and their TAdUTP‐inccorporating capacity.

Polymerase	3′–5′ Exonuclease activity	TAdUTP incorporation	Specifications	Family
iProof	yes	no	*Pyrococcus*‐like enzyme with a processivity‐enhancing Sso7d domain	B
OneTaq	yes	no	mixture of family A and B Taq and Deep Vent, respectively	A & B
Vent(exo‐)	no	yes	*Thermococcus litoralis*	B
PWO Superyield	yes	no	*Pyrococcus woesei*	B
Q5U	yes	no	high‐fidelity DNA polymerase fused to a processivity‐enhancing Sso7d domain	B
KOD XL	yes/no	yes	mixture of KOD and KOD(exo‐)	B
Pfu	yes	no	*Pyrococcus furiosus*	B
Therminator	no	no	9°N exo‐ variant	B

### TAdUTP alters physicochemical properties of oligonucleotides

Of note, the products provided by the modified nucleotide containing reaction mixture showed a decreased migration distance in PAGE indicating the incorporation of TAdUTP (Figure 2B). It has been previously shown that, modified nucleotide caused alteration in the physicochemical characteristics of DNA could be detected by melting curve analysis.^14^, ^15^ Thus, we amplified the three oligonucleotide templates by KOD XL either in the presence of dTTP or TAdUTP and determined the melting temperature of PCR product by gradually increasing the temperature during fluorescence measurement. The obtained data showed that substitution of dTTP with the studied modified nucleotide resulted in about 5–10 °C lower melting temperature (Table 2).

**Table 2:** Incorporation of TAdUTP results in PCR products with a decreased melting temperature.

	A1		A2		A3	
TAdUTP	−	+	−	+	−	+
*T*_m_±SD [°C]	88.64±0.03	78.74±0.07	89.60±0.03	82.2±0.05	89.00±0.03	83.55±0.05

### Amplification of oligonucleotide library by emulsion PCR minimalises the by‐product formation

**Figure 3:**
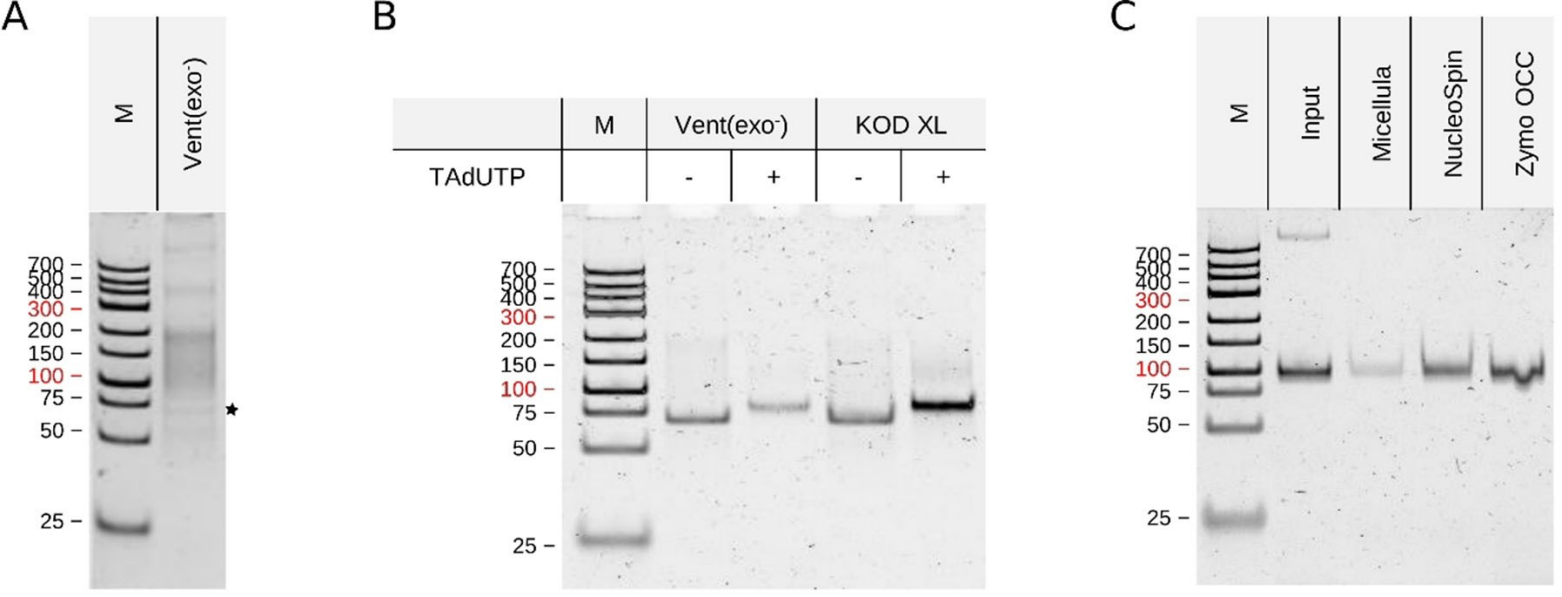
Aptamer library generation by PCR. A) Amplification of an aptamer library by using conventional, open PCR with Vent(exo‐). B) Amplification of an aptamer library by using water‐in‐oil emulsion PCR with Vent(exo‐) and KOD XL polymerases. C) Purification of modified oligonucleotides by using three commercially available DNA purification kits. In all cases, 5 μL of PCR products or eluted fractions were separated by PAGE and visualised with GelGreen dye.

It is recognised that the amplification of random oligonucleotide libraries of large sequence space is a challenging task.^16^ In concert with the previously published results, the amplification of our oligonucleotide library with traditional PCR either using dTTP or TAdUTP resulted in a multitude of by‐products of various sizes with hardly visible DNAs of expected base pair number (Figure 3A and Table S2). To overcome this obstacle, we assessed applicability of the micro‐reactor forming, water‐in‐oil emulsion PCR for amplification of an aptamer library with 10^14^ various sequences. According to the PAGE analysis of PCR products, by‐product formation decreased dramatically both with application of Vent(exo‐) and KOD XL DNA polymerases (Figure 3B). The replacement of dTTP with the modified nucleotide did not abolish the beneficial effect of emulsion PCR and, in harmony with our previous observation, resulted in a slower migration of DNA products. Surprisingly, PAGE analysis of completed PCRs indicated that KOD XL synthesised more DNA in the presence of TAdUTP than by the addition of dTTP.

The oil component of water‐in‐oil emulsion PCR has to be discarded prior to downstream processes; thus, membrane or bead based purifications are also included in the purification protocol of these reaction mixtures. The modified nucleotide containing DNA differs in its chemical properties from those of natural DNA as it was also shown by the altered electrophoretic mobility and melting temperature of our TAdUTP possessing DNAs. The available kits have been optimised for natural DNA purification; hence, it was essential to identify the most effective system for the isolation of modified nucleotide holding DNAs. To this end, we evaluated three commercially available DNA purification systems and found that the silica matrix based Zymo Oligo Clean and Concentrator kit provides the highest recovery of TAdUTP holding DNA (Figure 3C).

### Amplification by using the modified nucleotide neither increases the error rate nor decreases the sequence space

In order to reveal if application of TAdUTP has an adverse effect on the fidelity of Vent(exo‐) or KOD XL, we implemented a high‐throughput sequencing analysis of PCR products, which were produced by the amplification of the above described oligonucleotides (A1–A3, Table S1). Evaluation of the filtered reads indicated that the fidelity of both enzymes was similar to the previously published values and the addition of TAdUTP had a marginal effect on the error rate of DNA polymerisation (Tables 3, S1 and S3).

**Table 3:** The error rate of Vent(exo‐) and KOD XL, and ratio of unique sequences in the amplified library in presence of dTTP and TAdUTP, respectively.

	Vent(exo‐)		KOD XL	
TAdUTP	−	+	−	+
Error rate observed±SD [×10^−6^]	555±398	418±343	256±153	336±192
Published error rate [×10^−6^]	200^17^		3–4<Taq^10^	
Ratio of unique sequences to all filtered reads [%]	98.93	99.08	98.96	99.00

Next, we investigated the sequence composition of aptamer libraries, which were generated by water‐in‐oil emulsion PCR using the above two polymerases either in the presence of dTTP or TAdUTP. The unique sequences to all filtered reads ratio of PCR products demonstrated that sequence space of the generated aptamer library was close to the theoretical maximum (Tables 3 and S3). Furthermore, the difference was negligible if the oligonucleotides synthesised by dTTP and TAdUTP containing reaction mixtures were compared (Table 3). However, it should be noted that although the sequence spaces of the aptamer library were extremely similar whether they were generated using natural or modified nucleotide, the nucleotide composition of the libraries were different, that is, amplification of the libraries in the presence of TAdUTP resulted in a few percent over‐representation of guanosine and cytosine (Table 4).

**Table 4:** The nucleotide composition of the libraries amplified by either Vent(exo‐) and KOD XL in the presence of TAdUTP or dTTP.

	Vent(exo‐)		KOD XL	
TAdUTP	−	+	−	+
A [%]	27.10	22.50	25.00	22.30
T [%]	27.20	22.10	25.30	22.20
G [%]	22.70	27.80	24.50	27.90
C [%]	23.00	27.60	25.10	27.70

## Conclusion

In recent years, several research group adopted different methods to produce chemically modified aptamers of amino acid‐like properties to advance the practical applicability of aptamers. However, these approaches are not without shortcomings, as the introduction of new functional groups to the nucleotides might have an undesirable effect on the hydrogen bond formation of base‐pairs and unnatural nucleotides might not be readily accepted by thermostable DNA polymerases. These pitfalls could severely increase the error rate of DNA amplification and decrease the efficacy of polymerisation reaction, respectively. Considering the one of the essential steps of SELEX, that is, the target‐specific oligonucleotides are enriched by iterative PCRs, these drawbacks have to be minimalised to increase the success rate of aptamer selection. To the best of our knowledge, these putative effects of application of modified nucleotides have not been thoroughly studied yet. We implemented a comprehensive analysis by using 5‐indolyl‐AA‐dUTP, a commercially accessible amino acid‐like side chain holding nucleotide to identify the most auspicious thermostable DNA polymerase and reaction conditions for generating chemically modified aptamers and reveal the presumed adverse consequences of the usage of unnatural nucleotides. In concert with the previous findings, our data confirms that only 3’–5’ exonuclease deficient enzymes adapt nucleotides of amino acid‐like side chains. Although the 3’–5’ exonuclease activity deficient Vent(exo‐) and KOD XL enzymes accepted TAdUTP, the lack of this activity does not necessarily render the modified nucleotide incorporating capacity as the third studied 3’–5’ exonuclease domain lacking polymerase, Therminator, failed to use TAdUTP. The gel electrophoresis data showed that addition of modified nucleotides results in the alteration of physicochemical properties of PCR products and the lowered melting temperatures indicate that hydrogen bonds are also weakened in the TAdUTP holding dsDNAs. Considering this latter data, Vent(exo‐) and especially KOD XL seem to be barely sensitive for the inappropriately formed hydrogen bonds because both enzymes polymerised oligonucleotides by using TAdUTP, and the catalytic activity of KOD XL was not decreased by the replacement of dTTP with the modified nucleotide triphosphate. The aptamer library production and also the SELEX process are generally burdened by excessive by‐product formation that could be alleviated by applying water‐in‐oil emulsion PCR. Here, we presented that this approach could be productively leveraged for generating modified nucleotide libraries with a negligible amount of by‐product formation. Furthermore, the high‐throughput sequencing of individual PCR products and libraries obtained either by amplification of single oligonucleotides or random oligonucleotide libraries demonstrated that neither error rates of the enzymes nor sequence space of the library was changed by using TAdUTP. Collectively, these data demonstrate that amalgamation of water‐in‐oil emulsion PCR with the application of KOD XL thermostable polymerase could be the method of choice for the selection of chemically modified aptamers with amino acid‐like properties.

## Experimental Section

**Generation of oligonucleotides containing modified nucleotides**: Modified nucleotide containing oligonucleotides were acquired by amplifying a single‐stranded oligonucleotide template using different DNA polymerases. The modified nucleotide, 5‐[(3‐Indolyl)propionamide‐N‐allyl]‐2’‐deoxyuridine‐5’‐triphosphate (TAdUTP) was purchased from TriLink. Various DNA polymerases, such as OneTaq (New England Biolabs), Vent(exo‐) (New England Biolabs), Q5 U (New England Biolabs), Therminator (New England Biolabs), iProof (BioRad), PWO Superyield (Roche) and KOD XL (Toyobo) DNA polymerases were tested for their TAdUP incorporating capability. All PCR reactions were set up according to the manufacturers’ protocol by using 0.5 μM and 0.8 nM final primer and template concentrations, respectively. The applied primers and oligonucleotides were synthesised by IBA, the detailed sequences can be found in Table S1. Amplification conditions were: 3 min denaturation at 95 °C, 25 cycles of 95 °C for 30 s, 60 °C for 5 s, 72 °C for 30 s, and a final extension at 72 °C for 3 min. The PCR products were analysed by 10 % polyacrylamide gel electrophoresis. In all cases, 1 μL of GeneRuler Low Range DNA Ladder was used as molecular weight marker.

**Generation of modified nucleotide containing library**: The modified aptamer library was produced by either conventional or by water‐in‐oil emulsion PCR using Micellula DNA Emulsion & Purification Kit (Roboklon) for the latter. KOD XL and Vent(exo‐) DNA polymerases were tested in both arrangements. The PCR reactions were set up according to the manufacturers’ protocols by using the above described primer and template concentrations. In the case of the water‐in‐oil emulsion PCR, the PCR mixture was emulsified according to the manufacturer's protocol. The aptamer library and primer sequences are given Table S2 (IDT). The library was amplified by applying the above indicated thermal conditions.

**Recovery of PCR products from water‐in‐oil emulsion**: PCR products were either recovered using the Micellula DNA Emulsion & Purification Kit, NucleoSpin® Gel and PCR Clean‐up (Macherey‐Nagel) or Oligo Clean & Concentrator Kit (Zymo Research) according to the manufacturers’ protocols.

**Melting curve analysis by real‐time PCR**: The qPCR with melting curve analysis was carried out using QuantStudio 12 K Flex PCR System. The final concentration of 1X for SYBR™ Green I Nucleic Acid Gel Stain (Invitrogen), 50 nM of ROX (Thermo Scientific) was added to the reaction mixture containing KOD XL and its buffer, and 0.5 μM, 0.8 nM primer and template concentrations, respectively. The amplification conditions were: 3 min denaturation at 95 °C, 40 cycles of 95 °C for 30s, 60 °C for 60 s. Melting curve analysis was performed with gradually increasing the temperature from 60 °C to 95 °C, at 0.05 °C s^−1^. All measurements were performed in triplicate.

**NGS analysis of modified PCR products**: DNA concentration of each amplicon mix were determined by High‐Sensitivity D1000 ScreenTape system with 2200 Tapestation (Agilent Technologies) and Qubit dsDNA HS Assay Kit with Qubit 3.0 Fluorometer (Thermo Fisher Scientific).

For library construction, KAPA HyperPrep and Single‐Indexed Adapter Kit (Roche Diagnostics) was applied according to the manufacturer's protocol. The quality and quantity of the library was determined by using High Sensitivity DNA1000 ScreenTape system with 2200 Tapestation (Agilent Technologies) and Qubit dsDNA HS Assay Kit with Qubit 3.0 Fluorometer (Thermo Fisher Scientific), respectively. Pooled libraries were diluted to 1.6 pM for 2x80 bp paired‐end sequencing with 150‐cycle High Output v2.5 Kit on the NextSeq 550 Sequencing System (Illumina, San Diego, CA, USA) according to the manufacturer's instructions.

Raw sequenced reads were demultiplexed and adapter‐trimmed by using NextSeq Control Software, whilst FastQ Toolkit (Illumina) was applied to filter out reads having mean quality score less than 30. The average number of high‐quality reads used for further statistical analyses was ca. 370 000 per samle.

**NGS data analysis**: Forward and reverse reads were merged by using PEAR software,^18^ with a minimum 10 bp overlap.

Merged contigs were brought to uniform length and the program Usearch^19^ was used to determine the number of unique sequences, number of singletons (unique sequences that have exactly 1 copy), the most abundant unique sequence and nucleotide composition of random libraries. The original contigs were mapped back to non‐singleton unique sequences with 98 % identity threshold, to determine the clustering of sequences using the ratio of the mapped sequences to all sequences.

The error rate of Vent(exo‐) and KOD XL was calculated as the average of the ratio of unique reads to the total nucleotides read, using the NGS data derived from the sequencing of the three oligonucleotide template (Table S1) amplicons.

Ratio of unique sequences to all filter reads was calculated using the sequencing data of amplicons generated using the oligonucleotide library (Table S2) amplified by either Vent(exo‐) or KOD XL.

## Conflict of interest

The authors declare no conflict of interest.

## Supporting information

As a service to our authors and readers, this journal provides supporting information supplied by the authors. Such materials are peer reviewed and may be re‐organized for online delivery, but are not copy‐edited or typeset. Technical support issues arising from supporting information (other than missing files) should be addressed to the authors.

Supplementary
