# Supplementary material for: Analysis of Modified Nucleotide Aptamer Library Generated by Thermophilic DNA Polymerases
Source: Chembiochem. 2020 Jul 14;21(20):2939–44. doi: 10.1002/cbic.202000236 (PMC7689754; doi:10.1002/cbic.202000236)
Supplement: Supplementary file 1 — Supplementary [file CBIC-21-2939-s001.pdf]

ChemBioChem

Supporting Information

Analysis of Modified Nucleotide Aptamer Library Generated by Thermophilic DNA Polymerases

Krisztina Percze and Tamás Mészáros*

Table S1. Oligonucleotide and primer sequences

Name	Sequence (5'-3')	Primer sequences (5'-3')
**A1**	ATC CAG AGT GAC GCA GCA TGT GTT ATT TTT TCC TGT CCT GTC TGT TTA CGC ACT TGC CTG GAC ACG GTG GCT TAG T	Forward: ATC CAG AGT GAC GCA GCA , reverse: ACT AAG CCA CCG TGT CCA
**A2**	ATC CAG AGT GAC GCA GCA CCA GCC ACG CCA AGC CCC TTC TAA CTG CTG TGAGCT CAT CTG GAC ACG GTG GCT TAG T	Forward: ATC CAG AGT GAC GCA GCA , reverse: ACT AAG CCA CCG TGT CCA
**A3**	CAG TGA GTG ATG GTG AGG GTG AAT CGG TGT CGA CTA TTA AAT TAA GTT GTG GTT GTT CCC ACA CTG TCC ATA CAC G	Forward: CAG TGA GTG ATG GTG AGG G, reverse: CGT GTA TGG ACA GTG TGG G

Table S2. Library and primer sequences

Name	Sequence (5'-3')	Primer sequences (5'-3')
**Library**	ATC CAG AGT GAC GCA GCA NNN NNN NNN NNN NNN NNN NNN NNN NNN NNN GAG ATA TCG TGC TAC CGT GA	Forward: ATC CAG AGT GAC GCA GCA , reverse: TCA CGG TAG CAC GAT ATC TC

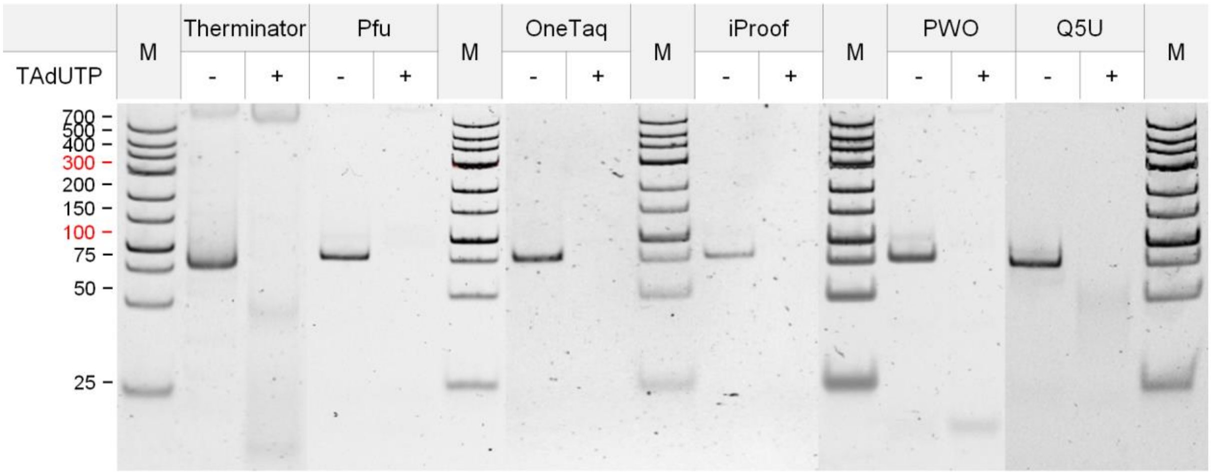

Figure S1. 5 µl of Therminator, Pfu, OneTaq, iProof, PWO Superyield and Q5U catalyzed reaction mixtures were separated on PAGE and visualised by GelGreen dye. The mixtures either contained only natural nucleotides (-) or dTTP was replaced by TAdUTP (+).

Table S3. NGS data obtained by analysing PCR products amplified by Vent(exo-) or KOD XL in the presence or absence of TAdUTP

**Template**	**A1**				**A2**				**A3**				**Aptamer library**			
**Enzyme**	**Vent(exo-)**		**KOD XL**		**Vent(exo-)**		**KOD XL**		**Vent(exo-)**		**KOD XL**		**Vent(exo-)**		**KOD XL**	
**PCR condition**	**TAdUTP**				**TAdUTP**				**TAdUTP**				**TAdUTP**			
	-	+	**-**	**+**	**-**	**+**	**-**	**+**	**-**	**+**	**-**	**+**	**-**	**+**	**-**	**+**
**Filtered reads**	263400	308905	318252	759835	322034	493453	386921	195415	335393	355436	371264	354417	403018	347986	493867	210661
**Unique reads**	4551	6177	3118	14186	18935	22657	9695	6061	6752	2362	3520	3043	398711	344800	488725	208546
**Max unique size**	118278	139942	159783	368350	90371	154320	6735	76416	137422	160568	166054	159134	17	8	21	17
**Singletons**	3467	4718	2364	10899	13254	15804	124973	4212	5050	1569	2629	2123	394442	341638	483686	206480
**Mapped to uniques (98%ID)**	260108	301011	315688	744850	296385	460716	377105	188327	327423	353290	367830	351445	8488	6327	10224	4312
**Mapped reads to uniqes ratio**	98.75%	97.44%	99.19%	98.03%	92.04%	93.37%	97.46%	96.37%	97.62%	99.40%	99.08%	99.16%	2.11%	1.82%	2.07%	2.05%
**Abundance**	258487	300086	315118	738159	235431	436797	285719	179572	327375	353246	367340	351410	19	10	33	27
**Error rate (58bp)**	2.98E-04	3.45E-04	1.69E-04	3.22E-04	1.01E-03	7.92E-04	4.32E-04	5.35E-04	3.53E-04	1.17E-04	1.66E-04	1.51E-04				
**Unique reads to filtered reads ratio**													98.93%	99.08%	98.96%	99.00%
